# Inflammatory choroidal neovascular membrane in posterior uveitis—pathogenesis and treatment

**DOI:** 10.4103/0301-4738.58467

**Published:** 2010

**Authors:** Narendra Dhingra, Susan Kelly, Mohammed A Majid, Claire B Bailey, Andrew D Dick

**Affiliations:** Department of Academic Ophthalmology, Bristol Eye Hospital, Lower Maudlin Street, Bristol, UK

**Keywords:** Choroidal neovascular membrane, immunosuppression, photodynamic therapy, posterior segment intraocular inflammation, posterior uveitis

## Abstract

Choroidal neovascular membrane (CNVM) formation is a well-documented sight-threatening complication of posterior segment intraocular inflammation (PSII). The aim of this article is to review the basic and clinical science literature on the pathogenesis of CNVM formation in PSII and to present results of a case series. We searched the literature using the mesh terms- inflammation, CNVM, age-related macular degeneration, immunosuppression, photodynamic therapy, steroids, vascular endothelial growth factors and posterior uveitis. Additionally, we evaluated the visual outcome of and clinical response to our standard treatment protocol involving a combination treatment for young patients with inflammatory CNVM. The development of CNVM in PSII is promulgated by infiltrating myeloid cells as well as choroidal and retinal myeloid cell activation, subsequent vascular endothelial growth factors, cytokine and chemokine production and complement activation acting in consort to mediate angiogenic responses. No clear standard of care currently exists for the treatment of inflammatory CNVM and various combinations have been tried. Using our combination treatment, visual acuity improved in four, stabilized in one and worsened in four patients. Though significant advances have occurred in the understanding of the pathogenesis and management of this condition, optimizing therapeutic regimens will require further well-constructed prospective cohort series.

Choroidal neovascular membrane (CNVM) formation is a well-documented sight-threatening complication of posterior segment intraocular inflammation (PSII). The development of CNVM results either directly from an inflammatory-mediated angiogenic drive and/or secondary to a degenerative disruption in the Bruch's membrane-retinal pigment epithelium (RPE) complex. The development of CNVM in PSII is promulgated by infiltrating myeloid cells as well as choroidal and retinal myeloid cell (microglial) activation, subsequent vascular endothelial growth factor (VEGF), cytokine and chemokine production and complement activation acting in consort to mediate angiogenic responses.

Based on their histology, Gass classified CNVMs into Type 1 and Type 2.[1] In Type 1, the subepithelial CNVM grows between the basement membrane of the RPE and the inner collagenous zone of Bruch's membrane. The CNVMs associated with punctate inner choroidopathy (PIC), presumed ocular histoplasmosis syndrome (POHS) and with other PSII are assumed to be Type 2 membranes; so called inflammatory membranes. In Type 2, the CNVM grows beneath the sensory retina, lying anterior to the RPE. Although the etiology is undoubtedly disparate between age-related Type I membranes and Type II membranes there remains a commonality to the extent of involvement of inflammation in their pathogenesis. The process of evolution and expression of CNVM could arguably be interpreted as representing a spectrum of inflammatory processes and thus, in this regard, we will review the impact of deregulated immunity and inflammation in the genesis of age-related CNVM and how with respect to intraocular inflammation, comparing and contrasting current evidence potentially highlights targets for improved treatment of inflammatory-induced CNVM.

Currently, traditional options available for managing inflammatory CNVM include observation, laser photocoagulation, local and systemic corticosteroids, and surgical removal; all with potential limitations.[2] To date, clinical trials on the treatment of inflammatory CNVMs associated with uveitis have been limited and mostly non-comparative. The best evidence on treatment of CNVM in PSII comes from the POHS study.[3] Whilst corticosteroids and immunosuppressive agents have an established role in treating PSII, their role is less certain, with less evidence for treatment of inflammatory CNVM. Nevertheless, some benefit has been demonstrated with photodynamic therapy (PDT) for inflammatory subfoveal CNVM. The short-term thrombogenic effects of PDT in combination with longer term anti-inflammatory effects of local and systemic immunosuppression may be more successful in achieving closure of these membranes than either treatment alone. The aim of this article is to review the basic and clinical science literature on the pathogenesis of CNVM formation in PSII and to present results of a case series.

## Methods

**Figure 1:**
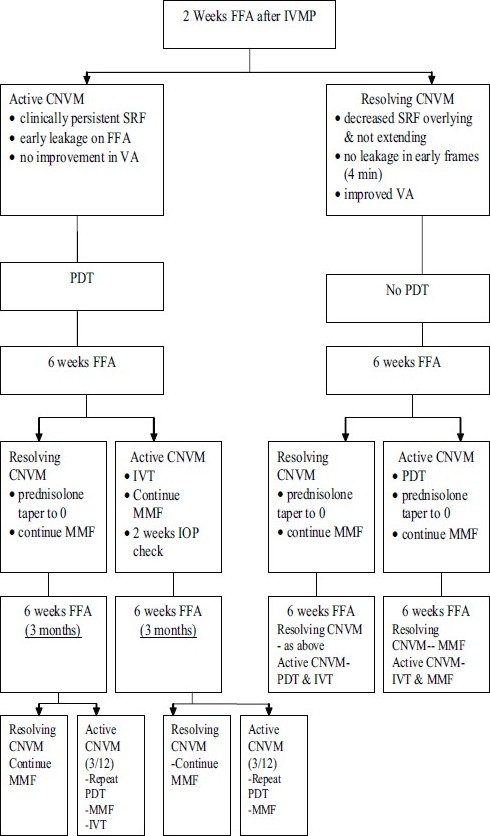
Algorithm for treatment used in the study (choroidal neovascular membrane; photodynamic therapy; intravitreal triamcinolone 4 mg/0.1 ml; mycophenolate mofetil 1 g po BD; fundus fluorescein angiography; subretinal fluid; visual acuity; intravenous methylprednisolone)

Based on current evidence we designed a treatment algorithm for the management of inflammatory CNVM [Fig. 1] using a combination of systemic steroids, immunosuppressives and PDT. In this review, we present results of our audit using this regimen and review the relevant basic and clinical science evidence on management of inflammatory CNVM. We searched the pubmed, medline, embase, Cochrane review using the mesh terms- inflammation, CNVM, age-related macular degeneration (AMD), immunosuppression, PDT, steroids, VEGF and posterior uveitis.

## Pathogenesis

Gass proposed the most widely accepted theory on formation of disciform lesions in ocular histoplasmosis. The process is believed to begin with focal infection of the choroid at the time of the initial benign infection. The focal infection resolves as an atrophic scar that disrupts the Bruch's membrane. The resulting break in the Bruch's membrane provides an opening in the barrier and subsequent tissue rearrangement through which the new vessels gain admittance into the subretinal space.[4]

### Why immunosuppress for the treatment of CNVM?

#### Role of Inflammation in pathogenesis of inflammatory CNVM

Recent studies provide mounting evidence that inflammation is pivotal in the pathogenesis of AMD.[5] Drusen contains complement components and complement regulators, immunoglobulins, and cells which may represent antigen-presenting cells (APC) and macrophages.[6] The possibility of systemic immune alongside local ocular (either structural or immunregulatory) dysregulation is compounded by studies where firstly significant associations were noted between elevated serum C-reactive protein (CRP) levels and polypoidal choroidal vasculopathy and neovascular AMD.[7]

Secondly, subjects with neovascular AMD had higher levels of serum elastin-derived peptides (S-EDPs) suggesting that higher levels of S-EDPs may increase the risk of conversion from early AMD to neovascular AMD.[8] Dysregulation of the extracellular matrix (ECM) plays an important role in the pathogenesis of AMD, whether mediated via inflammation or injury. Elastin is a fibrous protein constituent of the ECM, degradation of which may be detected by the presence of S-EDPs in the circulation. Chronic inflammatory infiltrates are observed in the inner choroid of donor eyes with AMD. Proteins associated with immune-mediated disease appear to accumulate in large quantities, and transcripts that encode a number of these molecules have been detected in retinal, RPE, and choroidal cells (large, veiled, MHC Class II^mid^ and small, MHC Class II^hi^ cells).[9] Dendritic cells, potent APC, have been reported to be intimately associated with drusen development. To this end, changes and turnover of the extracellular matrix and of Bruch's membrane have been likened to atherosclerosis, in which inflammatory phenomena occur and are also observed within Sorsby's dystrophy which shows changes in elastin of Bruch's membrane associated with TIMP-3 mutation.[10]

Interestingly, these findings are very similar to histological features of CNVM in patients with chronic posterior uveitis, such as idiopathic CNVM, and ocular histoplasmosis. Such similarity leads to infer that leukocytes and chronic chorioretinal inflammation are critical for the development of CNVM. Further good evidence supporting the role of innate inflammation in the pathogenesis of CNVM is the deposition of complement and immunoglobulin G (IgG) in RPE and choroid in patients with AMD. In a mouse model of laser-induced CNVM, C3 and membrane attack complex (MAC) are integral to the neovascular complex.[11] Depletion of complement using cobra venom factor inhibited the up-regulation of angiogenic factors, such as VEGF, bFGF, and TGF-β, and subsequent CNVM formation.[12] In C3-deficient mice, CNVM did not develop, further indicating the role of complement and inflammation in the pathogenesis of CNVM.

#### Role of Complement

The complement system is continuously activated at low levels in the normal eye and intraocular complement regulatory proteins tightly regulate this spontaneous complement activation to maintain complement activity at a level that promotes elimination of potential pathogens without damaging healthy tissue. Complement dysregulation leading to overactive complement activity can therefore cause immune-mediated ocular damage. Complement dysregulation has been well-characterized in experimental autoimmune anterior uveitis initiated with melanin-associated antigen, in which ocular specimens contain deposits of immune complexes such as IgG and complement component 3 (C3), as well as tumor necrosis factor (TNF)-α and interleukin (IL-1).[12]

**Figure 2:**
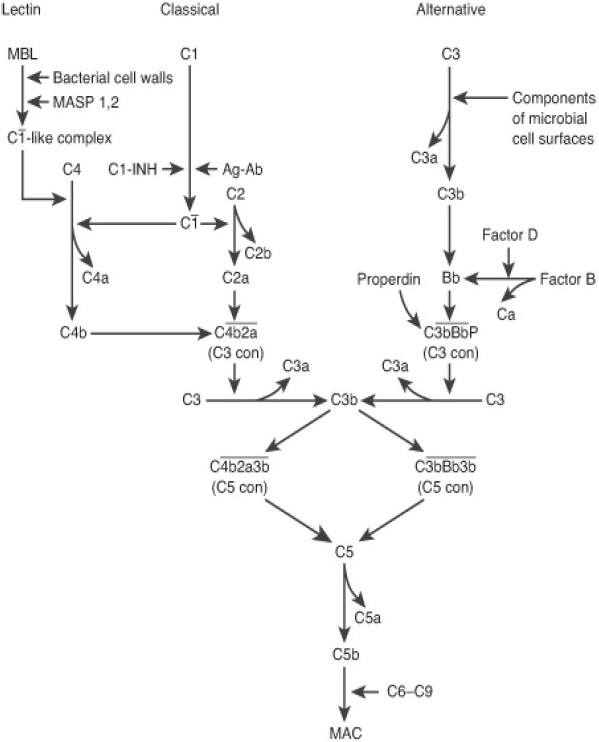
Complement pathway

Complement factor H (CFH) is an important inhibitor of the complement pathway. Activation of this pathway initiates a proteolytic cascade that releases chemokines and causes formation of a MAC, ultimately leading to cell lysis. Three enzyme cascades exist: the classical complement pathway, initiated by antigen-antibody complexes and surface-bound CRP; the lectin, turned on by microbial carbohydrates; and the alternative complement pathway, activated by surface-bound C3b. The pathways converge at the point in which C3 is cleaved into C3a and C3b by C3 convertase, which initiates C5 convertase, resulting in the formation of the MAC with the terminal components (C5b-C9).[13,14] CFH specifically inhibits the alternative complement cascade but also regulates the common pathway. It binds C3b and acts as a cofactor in the proteolysis of C3b by factor I, resulting in an inactive C3b molecule. This prevents the production of C3 convertase in the alternative cascade as well as the production of C5 convertase in the common pathway [Fig. 2]. The association between CFH and AMD emphasizes a support for the inflammatory pathogenesis of AMD and suggests that triggering the complement cascade in genetically predisposed individuals may promote development of AMD.

#### Role of Macrophages

Although classical ocular inflammatory diseases such as uveitis are mediated largely by T cells, macrophages also play an important role. Macrophages are the main components in granulomatous uveitis such as that associated with sarcoidosis, sympathetic ophthalmia, and Vogt-Koyanagi-Harada disease.[15] Macrophages are also one of the predominant inflammatory cells in anterior uveitis, experimental anterior uveitis and experimental autoimmune uveoretinitis (EAU).[15–17] Combating myeloid cell-mediated destruction of the retina during inflammation or neurodegeneration is dependent on the integrity of homeostatic mechanisms within the tissue that may suppress T cell activation and their subsequent cytokine responses. Success is dependent on the response of the resident myeloid-cell populations [microglia (MG)] to activation signals, commonly cytokines, and the control of infiltrating macrophage activation during inflammation, both of which appear highly programmed in normal and inflamed retina. The evidence that tissue CD200 constitutively provides down-regulatory signals to myeloid-derived cells via cognate CD200–CD200 receptor interaction supports inherent tissue control of myeloid cell activation. In the retina, there is extensive neuronal and endothelial expression of CD200. Retinal MG in CD200 knockout mice display normal morphology but unlike the wild-type mice, are present in increased numbers and express nitric oxide synthase 2, a macrophage activation marker, inferring that loss of CD200 or absent CD200R ligation results in “classical” activation of myeloid cells. Thus, when mice lack CD200, they show increased susceptibility to and accelerated onset of tissue-specific autoimmunity.[18]

Moreover, many proinflammatory cytokines (IL-1, IL-6, TNF-α) and chemokines that are produced by macrophages have been detected in various types of uveitic eyes.[19] Macrophages and the related multinucleate giant cells have similarly been demonstrated in histological specimens from patients with AMD, especially in regions of RPE atrophy, breakdown of Bruch's membrane, and CNVM.[20]

Overall, though various studies have highlighted several plausible mechanisms by which macrophages may have a pathogenic role for CNVM in human AMD, it still remains unknown whether macrophages accumulate near the CNVM because they play a causative role in CNVM or because they serve as an adaptive response against CNVM-associated pathology.

#### Role of Microglia (MG)

MG has been implicated in classical ocular inflammatory diseases such as uveitis. In early EAU, microglial migration to and activation at the photoreceptor end leads to the generation of TNF-α prior to infiltration of macrophages, suggesting that microglia might initiate EAU pathogenesis.[21] Whether MG initiate the recruitment of cells and cause amplification of inflammation is unclear. Another theory points to its migration to sites of injury as a regulator without evoking inflammatory damage or acting to suppress excessive inflammation and tissue damage.

#### Role of growth factors

The potential role of growth factors has been the subject of many studies. Pigment epithelial-derived growth factor (PEDF) appears to suppress neovascularization, while other factors, notably VEGF, stimulate blood vessel growth. VEGF plays critical roles in the development and progression of the vascularization system both in normal physiological and pathological conditions. VEGF expression is increased in CNVM in human exudative AMD patients and in animal models induced by laser treatment. PEDF is an endogenous molecule of ocular tissue cells that has active effects in inhibiting blinding complications of neovascular diseases of the eye. Intraocular delivery of PEDF in an adenoviral vector reduced angiogenesis beneath the retina in a laser model of CNVM.[22,23] PEDF is deficient in the vitreous of patients with CNVM due to AMD.[24] The declining vitreous and aqueous concentrations of PEDF with increasing age are more marked in AMD patients. PEDF as a treatment for AMD is currently being tested in animal models.

#### Supporting experimental evidence: Animal models

Progress in understanding the pathogenesis of AMD has been hampered by the lack of a suitable animal model. The laser-induced model has been used widely in studies evaluating the role of complement and macrophages in inducing CNVM. Studies in animal models of laser-induced CNVM demonstrate that CNVM complexes accumulate C3 and the C5b–9 MAC and that these components are capable of up-regulating the proangiogenic cytokine VEGF.

Mice deficient in *CCL2* or its receptor develop retinal degeneration with many pathological similarities to AMD. However, this animal model develops the AMD-like phenotype only in its senescent stage.[25] Aged mice knockouts for *CX_3_CR1* also sporadically develop drusen-like lesions, progressive accumulation of subretinal microglia, and photoreceptor degeneration.[26]

*CCL2*^−/−^/*CX_3_CR1*^−/−^ mice consistently develop retinal degeneration with many morphological and histological similarities to human AMD at six weeks of age.[27] More recently it was shown that several inflammatory proteins (F4/80, CD11b and C3d) which mediate or are involved in innate immune responses, are differentially expressed in the *CCL2*^−/−^/*CX_3_CR1*^−/−^ relative to the Wild Type controls.[28] Anti-retinal auto antibodies were also detected in the *CCL2*^−/−^/*CX_3_CR1*^−/−^ serum. These results support an immunological role in the retinal degeneration of the *CCL2*^−/−^/*CX_3_CR1*^−/−^ mice. The immune characteristics are similar to human AMD with complement deposition, MPC recruitment, and anti-retinal autoantibody detection.

### Clinical Review: Optimizing Therapy

Despite increasing our knowledge of immunopathogenesis from animal models and other experimental work; clinical translation has been less productive, relying on treatment paradigms extrapolated from therapies for age-related CNVM. Accordingly here we highlight seminal contributions, toward therapeutic interventions for inflammatory CNVM.

#### Natural history

Brown *et al*.[29] reported the development of CNVM in around 30% of patients with multifocal choroiditis with panuveitis (MCP) and around 40% of patients with punctate inner choroidopathy (PIC) over three years. The final visual acuity (VA) in all eyes was less than or equal to 20/200. At presentation CNVM is commoner in chronic low-grade disease (PIC) than in MCP (PIC, 76.9%; MCP, 22.7%).[30] A survey analysis of 77 patients with PIC[31] reported CNVM (69%) and subretinal fibrosis (56%) in the majority of participants in at least one eye. In a study of presumed ocular histoplasmosis (POHS), Kleiner *et al*.[32] showed that three-fourths of eyes with CNVM lost vision to a level of 20/100. At 39 months follow-up Olk *et al*.[33] revealed that 69% of POHS patients with CNVM had a final VA of ≤20/200.

#### Role of Laser

Thermal laser photocoagulation is not utilized for subfoveal CNVM as it results in immediate central vision loss. The macular photocoagulation study (MPS)[34] showed a clear benefit in terms of VA at three and five years post laser treatment for juxtafoveal CNVM in POHS compared with the non-treatment group. Nearly one-third of untreated eyes versus 8% of treated eyes lost greater than or equal to six lines of VA at five years' follow-up. However, this treatment is associated with persistent and recurrent CNVM in approximately one-third of cases. Additionally, enlargement of scars post treatment can result in vision loss and patients tend to be symptomatic with a paracentral scotoma.

#### Role of Surgery

Inflammatory CNVMs are classified by Gass as Type 2 membranes and they grow beneath the sensory retina and lie anterior to the RPE. Hence surgical removal allows preservation of the underlying RPE and choriocapillaries. Although initially successful, submacular surgery with CNVM extraction is associated with a high recurrence of subfoveal CNVM postoperatively.[35] Essex *et al*.[36] supported considering surgery in patients with acuities of 20/120 or less. There is limited information on macular translocation in the treatment of inflammatory CNVM.

#### Role of Corticosteroids

The visual results from the use of corticosteroids in inflammatory CNVM have been variable. Flaxel *et al*.[37] concluded from their series of 12 eyes with subfoveal CNVM due to MFC or PIC that systemic steroids resulted in stabilization of vision (83%).The course included 1 mg/kg/day of oral corticosteroid for three to five days followed by a taper over six to eight weeks. A further study[38] compared high-dose oral steroids (prednisolone) with a single subtenon's injection (triamcinolone) in 18 POHS patients. Stabilization of vision was reported in seven of the prednisolone group and five of the triamcinolone group. However, despite treatment, 72% had a final VA of less than 20/200.The final VA was similar in the two groups. In a study of 10 POHS patients with CNVM treated with intravitreal triamcinolone alone, Rechtman *et al*.[39] reported stable/improved vision in 80% of eyes and loss of vision in 20% of the treated eyes.

#### Role of PDT

PDT for inflammatory CNVM is of uncertain value. The verteporfin in ocular histoplasmosis study[40] showed an improvement in median VA from baseline of six letters at 24 months in 22 patients with subfoveal CNVM secondary to POHS. Forty-five percent of patients gained seven or more letters and 18% lost eight or more letters (mean number of treatments-3.9). Postelmans *et al*.[41] showed stable or improved vision in 81% of eyes with subfoveal CNVM secondary to PIC and POHS (mean number of treatments- 2.5). In another small case series[42] vision stabilized in all the eyes and the mean number of treatments was 2.8. Again, common to most studies, collateral damage to the surrounding tissue, hypoperfusion and RPE atrophy has all been recognized.

#### Combination therapy: PDT and corticosteroids

Combining PDT and corticosteroids has been shown to be more effective, with a lower re-treatment rate, as compared to PDT alone in inflammatory CNVM. In a study by Chan *et al*.[43] on idiopathic subfoveal and PIC-induced CNVM, 92.9% had stable or improved vision. The mean improvement in VA was 3.2 lines. The mean number of treatments during the one-year study period was 1.1. Fong *et al*.[44] reported 100% improvement in vision with PDT and systemic steroid for PIC CNVM with the average number of treatments being 2.0. Giovannini *et al*.[45] randomized 20 patients with idiopathic sub and juxtafoveal CNVM to either PDT alone or PDT and systemic steroid. The final VA at 20 months was significantly better in the combination group. In the PDT group the mean number of treatments was 2.3 compared to 1.2 in the combination group. The success of this combination treatment is attributed to both occluding the inflammatory CNVM and controlling intraocular inflammation.

#### Role of Anti-VEGF agents

A potential paradigm is that preceding retinal and choroidal neovascularization, a pro-inflammatory environment is created which activates both resident and tissue macrophages and infiltrating macrophages generating VEGF-A release.[46] VEGF is a potent mediator of pathological angiogenesis.

Adan *et al*.[47] demonstrated complete resolution of inflammatory CNVM in 100% of eyes treated with intravitreal avastin. Of a total of nine eyes followed for seven months, visual improvement occurred in eight eyes and stabilized in one eye. No patient had visual deterioration and a mean 1.3 injections/eye were needed. Chan *et al*.[48] reported visual and anatomic improvements in eyes with idiopathic CNVM and CNVM attributable to PIC and central serous choroidopathy. All 15 eyes had improvement in VA with a mean improvement of 2.9 lines at six months, as well as a reduction in central foveal thickness on ocular coherence tomography (OCT). Further studies are warranted to define its exact role as a monotherapy and the potential for combination treatment.

#### Role of immunosuppression

Immunosuppression has been used effectively to treat posterior uveitis and various white dot syndromes. Given that the inflammatory responses is central to the development of inflammatory CNVM, immunosuppressive agents are increasingly being used in its management.[49] Dees *et al*.[50] reported stabilization or improved vision in 53% and angiographic closure in 76% of inflammatory CNVM patients treated with corticosteroids and /or cyclosporin.

Successful management involves both control of intraocular inflammation and occlusion of the inflammatory neovascular membrane. No clear standard of care currently exists for the treatment of inflammatory CNVM.

### Combination therapy: PDT and Immunosuppression

#### Bristol Audit

We evaluated the visual outcome and clinical response of our standard treatment protocol involving combination immunosuppression, intravitreal triamcinolone (IVT) and PDT for young patients with inflammatory CNVM. Data was obtained retrospectively as part of routine audit of a treatment algorithm in nine patients with active inflammatory peripapillary, subfoveal and juxtafoveal CNVM. In all cases, active choroidal neovascularization was diagnosed clinically by the presence of subretinal fluid and hemorrhage. This was confirmed by optical coherence tomography (OCT, Stratus III, Zeiss, UK) and scanning laser ophthalmoscopy-based fundus fluorescein angiography (FFA) within one week of presentation.

As per the protocol [Fig. 1] patients were admitted and treated with three days of pulse intravenous methylprednisolone (IVMP, 1 g daily). Immunosuppression with mycophenolate mofetil (MMF 1 g BD) was commenced on the day of admission, if baseline bloods including full blood count, urea, electrolytes, creatinine and liver function tests (FBC, UEC and LFT) were within normal limits. Blood pressure, venous glucose levels and weight were also routinely monitored. Patients were discharged after three days of IVMP on a tapering dose of oral systemic steroid. The initial dose was 1 mg/kg/day for three to five days and was reduced to 0 mg over a six-week period.

The FFA was repeated at two weeks and a decision was made at this time as to whether the CNVM was still active or resolving. This decision was based on VA (stable/ improved), the amount of subretinal fluid and the amount of fluorescein leakage. Active CNVM was treated with PDT and resolving lesions were monitored. Routine blood monitoring (FBC, UEC, and LFT) was maintained for patients on MMF. FFA was repeated one month later and disease activity was reassessed as before. If the lesion remained active, IVT (4 mg in 0.1 ml), was administered. In cases where the lesion was resolving post PDT, no steroid was warranted. If the membrane was considered active and PDT had not been administered at two weeks, it was given at six weeks. For those lesions that continued to resolve, no additional treatment was given. The next scheduled FFA was at three months and persistent activity, despite prior PDT at two weeks and IVT at six weeks, was re-treated with PDT. Those lesions that were resolving post PDT and IVT were monitored.

For the group who had only received PDT at two weeks and were active at three months, both IVT and PDT were given. This combination treatment was also administered to those lesions that were active at three months who had no previous treatment. IVT alone was injected if the CNVM was active at three months and PDT had been given at six weeks. Throughout this timeframe, MMF was continued at 1g PO BD. In all patients, PDT was performed according to the TAP guidelines.[51] IVT was injected in theatre under aseptic conditions.

## Results

**Figure 3:**
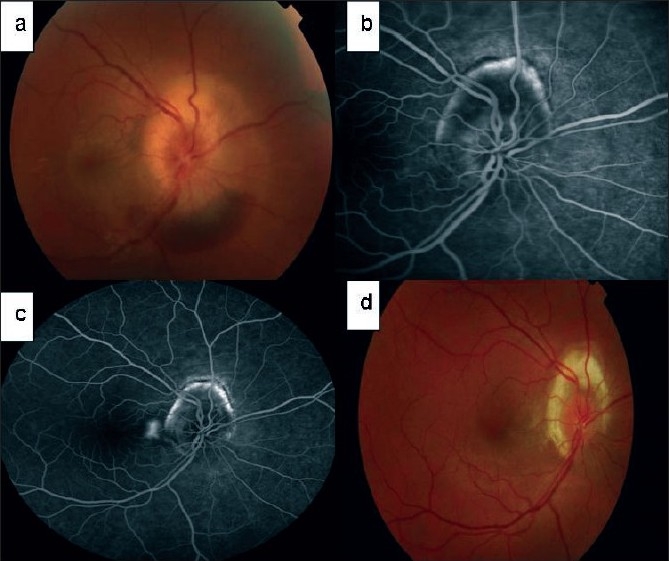
Patient with idiopathic panuveitis with peripapillary choroidal neovascular membrane [initial fundus photograph showing swollen optic nerve head with inferior subretinal hemorrhage (a), fundus fluorescein angiography two weeks after intravenous methylprednisolone and commencement of mycophenolate mofetil (b), fundus fluorescein angiography on the day of photodynamic therapy shows only a small area of active choroidal neovascular membrane temporal to the optic disc, fundus photograph at 13 months shows peripapillary atrophy and fibrosis (b)]

Of the nine patients (four males and five females), four had CNVM secondary to PIC, four were idiopathic and one secondary to POHS. The median age at presentation was 32 years (range 22 –62). The lesions were classified as peripapillary (two), juxtafoveal (four) and subfoveal (three) CNVM. The median lesion size was 2085 μm (range 800–3000 μm). The median follow-up was 13 months (range 2 –23). Improvement or stabilization was defined as the proportion of eyes with fewer than 15 letters (approximately three lines) of VA loss as devised in the TAP study. The study showed, albeit with variable follow-up that VA improved in four out of nine patients, stabilized in one and worsened in four patients. Only one patient had severe vision loss due to choroidal hypoperfusion. The median VA at presentation was 70 ETDRS letters and at final follow-up was 75 ETDRS letters. CNVM activity reduced in 100% of patients by the second month [Table 1, Fig. 3].

**Table 1:** Results using the algorithm (intravenous methylprednisolone; photodynamic therapy; intravitreal triamcinolone 4 mg/0.1 ml; visual acuity; choroidal neovascular membrane; juxtafoveal, subfoveal, peripapillary; punctate inner choroidopathy; presumed ocular histoplasmosis syndrome)

Age	Sex	Diagnosis	Type/size CNV (μ)	IVMP (n)	PDT (n)	IVT	Baseline VA (ETDRS)	Final VA	Follow-up (months)
29	F	PIC	JF/1200	1	1	0	60	85	11
32	F	PIC	SF/2100	1	1	1	35	35	12
34	F	PIC	JF/860	1	0	0	85	80	02
27	F	PIC	JF/2788	3	3	0	70	60	23
27	M	Idiopathic	SF/1500	1	2	0	55	75	07
42	M	Idiopathic	JF/1200	1	3	0	75	30	20
62	F	Idiopathic	PP/3000	3	3	0	85	75	16
22	M	Idiopathic	PP/3000	2	0	0	70	75	13
50	M	POHS	SF/800	1	3	1	70	75	14

No complications were recorded in relation to either PDT or the intravitreal injection. One patient had gastrointestinal upset on MMF. The hematological and biochemical parameters remained within normal limits in all the patients.

## Discussion

Traditional options available for managing inflammatory CNVM include observation, laser photocoagulation, local and systemic corticosteroids, and surgical removal; all with potential limitations.[2] To date, clinical trials on the treatment of inflammatory CNVMs associated with uveitis have been limited and mostly non-comparative. While corticosteroids and immunosuppressive agents have an established role in treating PSII, their role is less certain for the treatment of inflammatory CNVM. Nevertheless, some benefit has been demonstrated with PDT for inflammatory subfoveal CNVM. The short-term thrombogenic effects of PDT in combination with longer term anti-inflammatory effects of local and systemic immunosuppression may be more successful in achieving closure of these membranes than either treatment alone.

Our treatment algorithm combines the use of all the available modalities including steroids (local and systemic), PDT and immunosuppression. Corticosteroids inhibit the proliferation of vascular endothelial cells by suppressing the actions of inflammatory cells and their secretion of proangiogenic mediators. Corticosteroids decrease vascular permeability by stabilizing the basement membrane of the CNVM, and this may prevent vascular budding. Immunosuppression is required to dampen the chronic inflammatory drive.

In our series the CNVM activity reduced in all the patients and five patients noted an improvement/stabilization of vision. The others experienced a reduction in VA by one line (three) and by eight lines (one). Our results are comparable to PDT alone and combination studies (PDT and corticosteroids) mentioned previously. Common to most studies and ours is the collateral damage to the surrounding tissue, hypoperfusion and RPE atrophy from PDT leading to reduction in final VA. The caveats of this study include: retrospective audit, small numbers and variable follow-up. Whilst the rate of events such as resolution of CNVM and changes in VA can overcome the inherent misinterpretation of conclusions drawn from presenting data as for example final VA when there is variable follow-up for larger retrospective series,[52] there remains little bias in this study as we present core data on small patient numbers, as shown in Table 1. What is evident is that VA improvement did not parallel the success in CNVM resolution and activity.

Although there have been some promising early results with anti-VEGF agents in treating inflammatory CNVM, the data is limited and it remains to be elucidated whether a combination of anti-VEGF agents with other treatments will result in better visual outcomes. From our small series we would not like to draw any firm conclusions with regards to best treatment for inflammatory CNVM and the decision is left to the treating ophthalmologists, and may be made clearer in the future with studies of anti-VEGF therapy for such conditions.

To summarize, significant advances have recently occurred in the understanding of the pathogenesis and management of this condition. Optimizing therapeutic regimes will require further well-constructed prospective cohort series. Adequately powered randomized clinical trial data will be difficult to obtain given the rarity of the condition.
